# An acid tale of prion formation

**DOI:** 10.7554/eLife.22256

**Published:** 2016-11-29

**Authors:** Mick F Tuite

**Affiliations:** Kent Fungal Group, School of Biosciences, University of Kent, Canterbury, United KingdomM.F.Tuite@kent.ac.uk

**Keywords:** *Dekkera bruxellensis*, *Staphylococcus gallinarum*, lactic acid, prion, catabolite repression, molecular communication, *S. cerevisiae*

## Abstract

Some bacteria use lactic acid to communicate with yeast cells.

**Related research article** Garcia DM, Dietrich D, Clardy J, Jarosz DF. 2016. A common bacterial metabolite elicits prion-based bypass of glucose repression. *eLife* **5**:e17978. doi: 10.7554/eLife.17978

The transformation of grape juice into an appetising alcoholic beverage is not as straightforward as one might imagine. The yeast *Saccharomyces cerevisiae* is responsible for fermenting the sugars naturally found in grapes to make ethanol and carbon dioxide. Yet grapes, like many fruits, also contain L-malic acid, which has a sour taste, so the winemaker must trigger a process known as malolactic fermentation to turn the offending L-malic acid into the more palatable and ‘softer’ tasting L-lactic acid.

A variety of bacteria carry out the malolactic fermentation. Such bacteria may already be present on the skin of the grapes or the winemaker can add them after the alcoholic fermentation has taken place. *Oenococcus oeni* is the bacterium of choice for winemakers, partly because – unlike many other bacteria that produce lactic acid – it does not inhibit the ability of the yeast to ferment sugars.

Louis Pasteur first noted that lactic acid-producing bacteria are often associated with failed wine fermentations over 140 years ago (Pasteur, 1873; Alexandre et al., 2004). Yet, we are only now beginning to understand the complexity of the interplay between these bacteria and yeast during winemaking, thanks largely to the work of the late Susan Lindquist (who died only last month), and Daniel Jarosz, who is now at Stanford University School of Medicine. Now, in eLife, David Garcia of Stanford, David Dietrich and Jon Clardy of Harvard Medical School, and Jarosz report that lactic acid produced by certain bacteria can trigger the formation of the [*GAR*^+^] prion in fermenting yeast (Garcia et al., 2016).

Prions are made when proteins undergo a change in conformation and activity that they can sustain themselves and help other proteins to adopt. This in turn gives rise to a heritable form of the protein (known as the amyloid form) that assembles into clumps. However, unlike other yeast prions, the transmission of [*GAR*^+^] from cell to cell is not associated with a heritable amyloid. Rather, two other proteins contribute to the formation of the [*GAR*^+^] prion in yeast cells (Brown and Lindquist, 2009). But what has the [*GAR*^+^] prion got to do with lactic acid and failed wine fermentations?

The [*GAR*^+^] prion triggers a remarkable reprogramming of the carbohydrate metabolism in the host cell. By overriding an ancient mechanism that promotes the breakdown of glucose over other sources of carbon, [*GAR*^+^] cells are able to metabolise a wide range of other carbon sources, even when glucose is present (Brown and Lindquist, 2009). This is bad news for the winemaker because [*GAR*^+^] cells generate less ethanol, which aids the growth of lactic acid-producing bacteria (Figure 1).

**Figure 1.**
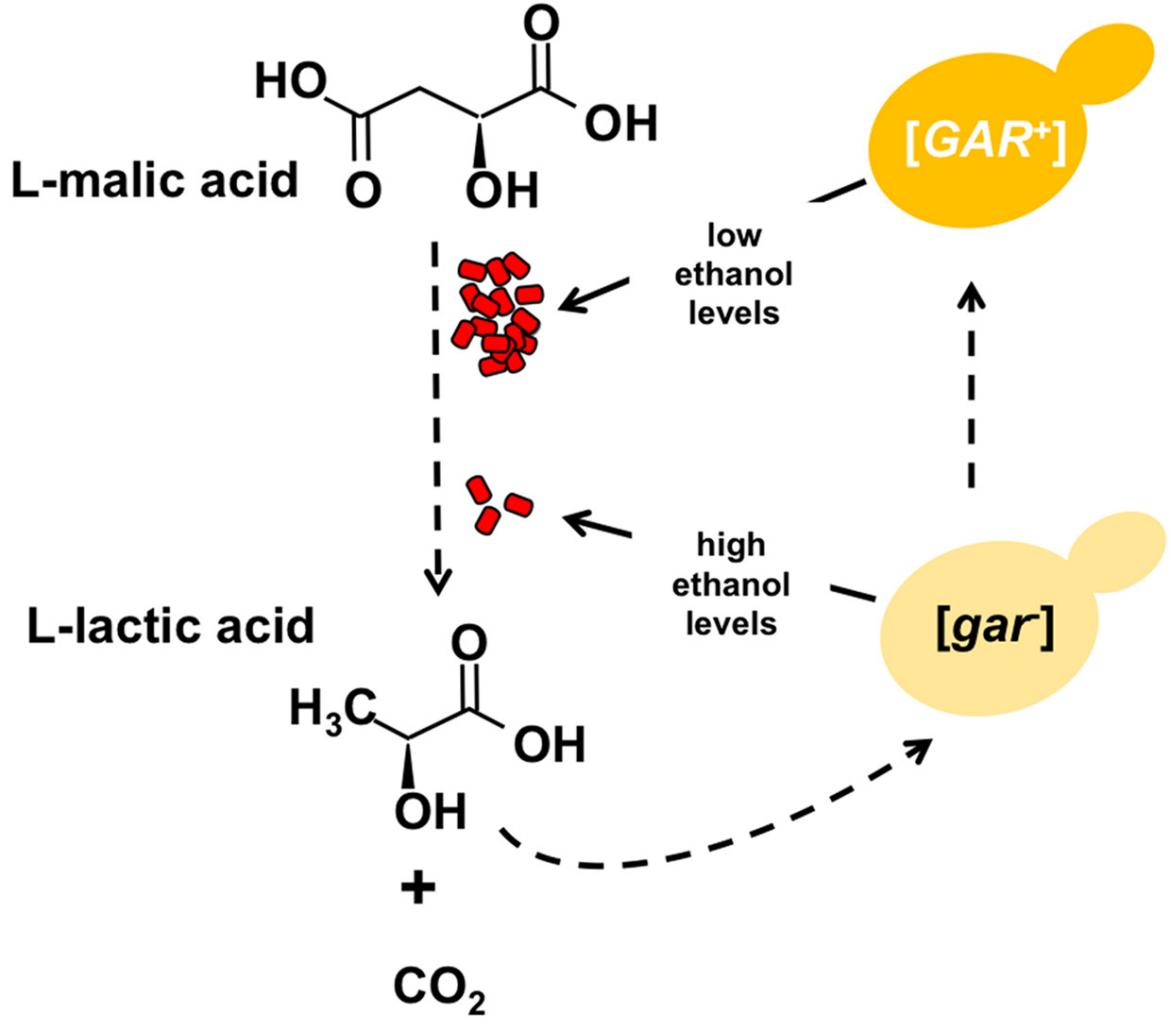
Certain bacteria communicate with yeast cells via lactic acid. L-malic acid (C_4_H_6_O_5_) is a natural component of grapes and other soft fruits, and it is decarboxylated by certain bacteria (red) during malolactic fermentation to produce L-lactic acid (C_3_H_6_O_3_) and carbon dioxide (CO_2_). The L-lactic acid can then induce the formation of the [*GAR*^+^] prion from the [*gar^-^*] protein in yeast cells (yellow). Cells carrying this prion (dark yellow) produce less ethanol from the fermentation of sugars, which in turn provides an environment in which the bacteria can flourish. Winemakers try to promote the production of ethanol and reduce levels of malic acid.

Earlier studies by Jarosz and Lindquist found that a wide range of bacteria secrete a chemical messenger (with a low molecular weight) that induces the appearance of [*GAR*^+^] in several different yeast species (Jarosz et al., 2014a, 2014b). Since this messenger was resistant to digestion by enzymes, researchers assumed that it was a metabolite. Garcia et al. now report that L-lactic acid can serve as this messenger.

This is not to say that L-lactic acid is the only messenger. Moreover, it is striking that a number of lactic acid-producing bacteria do not induce the [*GAR*^+^] prion. These include *O. oeni* (Jarosz et al., 2014b), whose inability to induce the prion may well contribute to its suitability for wine production. It has been known for over 50 years that the interactions between yeast and the bacteria involved in malolactic fermentation depend on the combinations of species present (Fornachon, 1968), and now we better understand why such variation exists.

The physical structure of the [*GAR*^+^] prion and the method by which lactic acid induces its formation remain to be established. However, the new communication mechanism discovered by Garcia et al. is important because it is beneficial to both yeast and bacteria in their native environments, and because it finally explains an observation first made by Pasteur over 140 years ago.
